# Universal Nature of Drug Treatment Responses in Drug-Tissue-Wide Model-Animal Experiments Using Tensor Decomposition-Based Unsupervised Feature Extraction

**DOI:** 10.3389/fgene.2020.00695

**Published:** 2020-08-20

**Authors:** Yh. Taguchi, Turki Turki

**Affiliations:** ^1^Department of Physics, Chuo University, Tokyo, Japan; ^2^Department of Computer Science, King Abdulaziz University, Jeddah, Saudi Arabia

**Keywords:** tensor decomposition, unsupervised learning, gene expression profiles, gene selection, drug treatment

## Abstract

Gene expression profiles of tissues treated with drugs have recently been used to infer clinical outcomes. Although this method is often successful from the application point of view, gene expression altered by drugs is rarely analyzed in detail, because of the extremely large number of genes involved. Here, we applied tensor decomposition (TD)-based unsupervised feature extraction (FE) to the gene expression profiles of 24 mouse tissues treated with 15 drugs. TD-based unsupervised FE enabled identification of the common effects of 15 drugs including an interesting universal feature: these drugs affect genes in a gene-group-wide manner and were dependent on three tissue types (neuronal, muscular, and gastroenterological). For each tissue group, TD-based unsupervised FE enabled identification of a few tens to a few hundreds of genes affected by the drug treatment. These genes are distinctly expressed between drug treatments and controls as well as between tissues in individual tissue groups and other tissues. We also validated the assignment of genes to individual tissue groups using multiple enrichment analyses. We conclude that TD-based unsupervised FE is a promising method for integrated analysis of gene expression profiles from multiple tissues treated with multiple drugs in a completely unsupervised manner.

## Background

Drug design is a time-consuming and expensive process. Multiple coordinated experimental efforts, involving large-scale trial-and-error methods, are required to investigate new compounds. In general, this is due to the inherent difficulties in identifying novel therapeutic targets such as genes that cause disease. Even where potential target genes are identified robustly, it is difficult to find drug candidate compounds that successfully bind to the proteins they encode.

Computer-based methods have been introduced in an attempt to shorten the period of drug development and to reduce the expenses involved. The two major computer-aided drug design strategies are ligand-based drug design (LBDD) and structure-based drug design (SBDD). LBDD has various advantages including less required computational resources and better success rates for drug design. However, it also has the disadvantage of limited ability to find drug candidate compounds with low structural similarity to known drugs. To compensate for the weaknesses of LBDD, SBDD shows a greater ability to find drug candidate compounds lacking in structural similarity with known drugs. This is because SBDD tries to screen drug candidate compounds by investigating whether these can bind to target proteins. The weak point of SBDD is that it requires massive computational resources, and this prevents its application to large-scale screening, in which candidate drug compounds often number several million.

Considering the relatively low cost of obtaining gene expression profiles, a third computer-aided strategy has been proposed: gene expression profile-based drug design. In this strategy, the gene expression profiles of tissues/cell lines treated with candidate drug compounds are collected. The collected profiles are then compared with those of tissues/cell lines treated with known drug compounds. If the candidate drug compounds share a gene expression profile to some extent with known drug compounds, they are identified as having therapeutic potential against target diseases/proteins.

Some databases have been established to assist gene expression profiling for drug design. For example, chemical checker (Duran-Frigola et al., 2020) includes gene expression in computer-aided drug design, whereas PharmacoDB (Smirnov et al., 2017) is fully implemented to consider the dose dependence of drug-treated cell lines for drug design. Many papers have been published on the use of gene expression profiles for computer-aided drug design (Chengalvala et al., 2007; Bates, 2011). For instance, Huang et al. (2019) used combinatorial analysis of drug-induced gene expression for cancer drugs, which were then experimentally confirmed *in vitro*. Lee et al. (2017) proposed DeSigN, a robust and useful method for identifying candidate drugs using an input gene signature obtained from gene expression analysis. Kim et al. (2019) performed computational drug repositioning for gastric cancer using reversal of gene expression profiles, and De Wolf et al. (2018) analyzed high-throughput gene expression profiles to identify similarities between drugs and to predict compound activity. Hodos et al. (2017) tried to fill in missing gene expression observations in cells treated with drugs by predicting cell-specific drug perturbation profiles using available expression data from related conditions. Pabon et al. (2018) predicted protein targets for drug-like compounds using transcriptomics. In contrast, Liu et al. (2017) performed comparative analysis of genes that are frequently regulated by drugs based on connectivity to map transcriptome data.

In contrast to these successful applications of gene expression profile analysis to computer-aided drug design, it is unclear how individual gene expression is affected by drug treatment. First, the number of genes expressed in a dose dependent-manner is as large as the number of genes expressed. Thus, it is not easy to invent a useful method to integrate and understand the dose dependent-genes pertaining to individual gene expression profiles. For example, Lukačišin and Bollenbach (2019) employed principal component analysis (PCA) to integrate the dose dependence of gene expression profiles upon combinatorial drug treatment. They reported a convex (not monotonic) dependence on dose density and identified this as evidence of the cooperative effects of dual drug treatments. Nevertheless, convex dependence on dose was reportedly observed in a single drug treatment if tensor decomposition (TD) was employed to integrate multiple gene expression profiles of cell lines treated with a single drug (Taguchi, 2019). Thus, it is primarily important to identify an effective method that can integrate numerous gene expression profiles of tissues/cell lines treated with drugs.

Recently, Kozawa et al. (2020) used the gene expression profiles of mouse tissues treated with drugs to predict human clinical outcomes. In this paper, we applied TD-based unsupervised feature extraction (FE) to the gene expression profiles used in their study and attempted to identify the changes in gene expression profiles of mouse tissues treated with individual drugs.

## Methods and Materials

**Figure 1:**
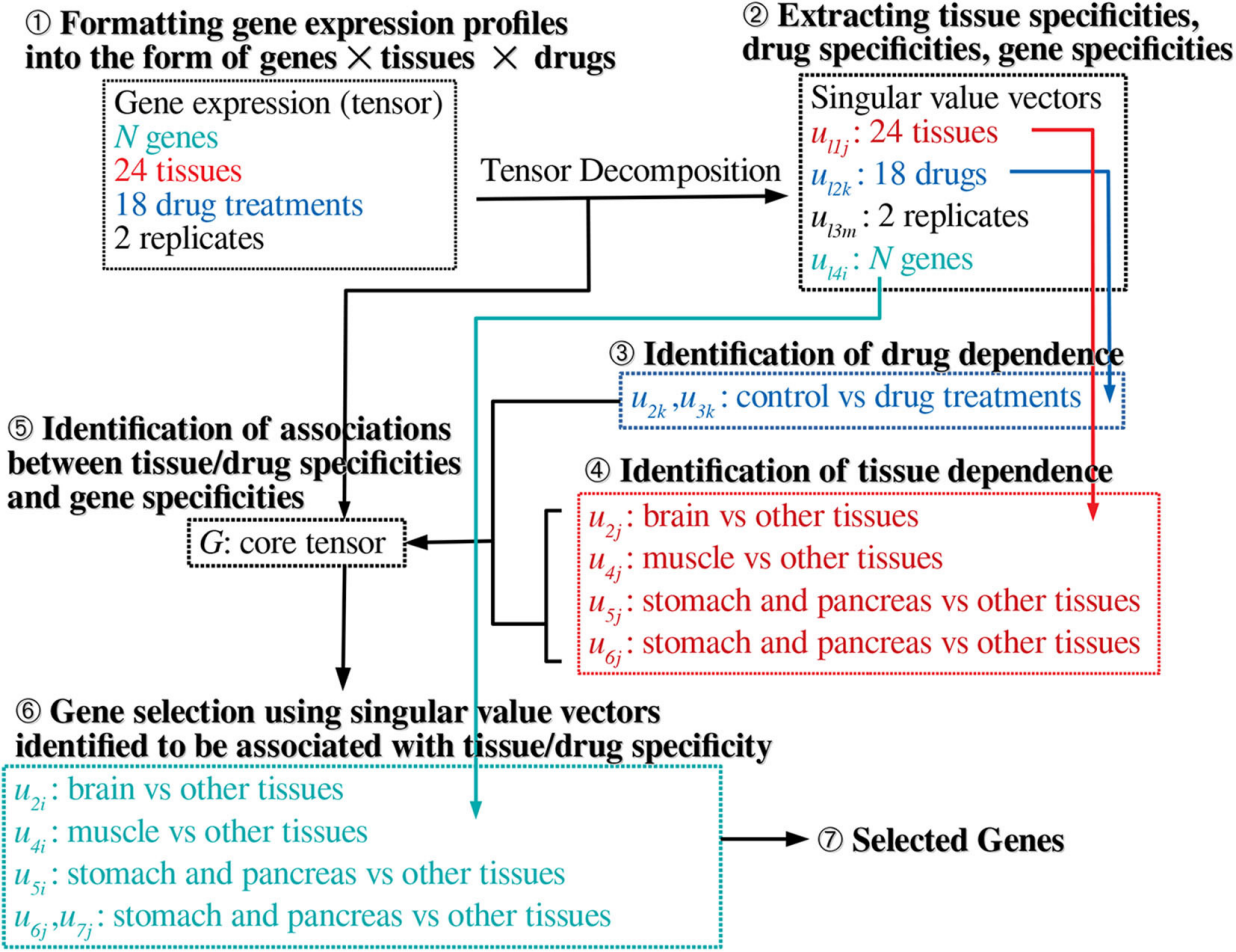
Schematic of the analysis performed in this study.

Figure 1 shows the flow chart of analysis.

###  Gene Expression Profiles

The gene expression profiles used in this study were downloaded from the gene expression omnibus (GEO) with GEO ID GSE142068. Twenty four profiles named “GSE142068_count_XXXXX.txt.gz” were downloaded, where “XXXXX” indicates one of the 24 tissues, i.e., AdrenalG, Aorta, BM (Bone marrow), Brain, Colon, Eye, Heart, Ileum, Jejunum, Kidney, Liver, Lung, Pancreas, ParotidG, PituitaryG, SkMuscle, Skin, Skull, Spleen, Stomach, Spleen, Thymus, ThyroidG, and WAT (white adipose tissue), which were treated with 15 drugs: Alendronate, Acetaminophen, Aripiprazole, Asenapine, Cisplatin, Clozapine, Clozapine, Empagliflozin, Lenalidomide, Lurasidone, Olanzapine, Evolocumab, Risedronate, Sofosbuvir, and Teriparatide, and Wild type (WT).

###  TD-Based Unsupervised FE

For applying TD-based unsupervised FE (Taguchi, 2020) to the downloaded gene expression profiles, they must be formatted as a tensor. In this analysis, they were formatted as tensor, $x_{ijkm}\in {\mathbb{R}}^{N\times 24\times 18\times 2}$, for *N* genes, 24 tissues, 18 drug treatments, and two replicates. Then, the HOSVD (Taguchi, 2020) algorithm was applied to *x*_*ijkm*_ and we obtained TD

(1) $$\begin{array}{l} x_{ijkm}=\underset{\ell_{1}\ell_{2}\ell_{3}\ell_{4}}{\sum }G\left(\ell_{1},\ell_{2},\ell_{3},\ell_{4}\right)u_{\ell_{1}j}u_{\ell_{2}k}u_{\ell_{3}m}u_{\ell_{4}i} \end{array}$$

where *G* ∈ ℝ^*N*× 24 × 18 × 2^ is the core tensor, $u_{\ell_{1}j}\in {\mathbb{R}}^{24\times 24}$, $u_{\ell_{2}k}\in {\mathbb{R}}^{18\times 18}$,$u_{\ell_{3}m}\in {\mathbb{R}}^{2\times 2}$, and $u_{\ell_{4}i}\in {\mathbb{R}}^{N\times N}$, represents singular value matrices that are also orthogonal matrices. *x*_*ijkm*_ is considered to be standardized as $\underset{i}{\sum }x_{ijkm}=0$ and $\underset{i}{\sum }x_{ijkm}^{2}=N$.

Mathematically, Equation (1) aims to decompose the dependence of *x*_*ijkm*_ upon *i, j, k, m* into a series of products among *u*_ℓ_1_*j*_, *u*_ℓ_2_*k*_, *u*_ℓ_3_*m*_, and *u*_ℓ_4_*i*_, each of which is supposed to represent the dependence on *i, j, k, m*. As it is unlikely that a single product of *u*_ℓ_1_*j*_, *u*_ℓ_2_*k*_, *u*_ℓ_3_*m*_, and *u*_ℓ_4_*i*_ can reproduce *x*_*ijkm*_, we need to consider various combinations of *u*_ℓ_1_*j*_, *u*_ℓ_2_*k*_, *u*_ℓ_3_*m*_, and *u*_ℓ_4_*i*_, where those associated with distinct ℓ_1_, ℓ_2_, ℓ_3_, ℓ_4_ are supposed to be associated with distinct dependence on *i, j, k, m*. Then, the products of *u*_ℓ_1_*j*_, *u*_ℓ_2_*k*_, *u*_ℓ_3_*m*_, and *u*_ℓ_4_*i*_, must be summed up with the weight of *G* to reproduce *x*_*ijkm*_. Biologically, we cannot expect that *u*_ℓ_1_*j*_, *u*_ℓ_2_*k*_, *u*_ℓ_3_*m*_, and *u*_ℓ_4_*i*_ can represent the biological aspect because Equation (1) is simply a mathematical hypothesis; therefore, their association with a biological aspect after performing TD needs to be validated.

To understand how gene expression profiles are altered by drug treatment in a tissue-group-wide manner, we first need to investigate *u*_ℓ_1_*j*_, *u*_ℓ_2_*k*_, and *u*_ℓ_3_*m*_. After identifying which ℓ_1_, ℓ_2_, and ℓ_3_ are biologically interesting, we select ℓ_4_ associated with *G*(ℓ_1_, ℓ_2_, ℓ_3_, ℓ_4_) that have the largest absolute values with fixed ℓ_1_, ℓ_2_, and ℓ_3_, because *u*_ℓ_4_*i*_ associated with such ℓ_4_ is supposed to represent the weight of gene *i* that is expressed in association with *j, k, m* dependence represented by the selected *u*_ℓ_1_*j*_, *u*_ℓ_2_*k*_, *u*_ℓ_3_*m*_.

Using the identified *u*_ℓ_4_*i*_, the *P*-value, *P*_*i*_, is attributed to gene *i* as

(2) $$\begin{array}{l} P_{i}=P_{\chi^{2}}\left[>{\left(\frac{u_{\ell_{4}i}}{\sigma_{\ell_{4}}}\right)}^{2}\right] \end{array}$$

where $P_{\chi^{2}}\left[>x\right]$ is the cumulative probability of χ^2^ distribution and σ_ℓ_4__ is the standard deviation. Here, we assume that *u*_ℓ_4_*i*_ obeys a Gaussian distribution with zero mean because $\underset{i}{\sum }x_{ijkm}=0$. *P*_*i*_ is corrected via the BH criterion (Burgos et al., 2014) and *I*, a set of genes *i* associated with adjusted *P*-values less than 0.01, is selected. For a more detailed explanation of TD-based unsupervised FE, see the recently published monograph (Taguchi, 2020).

### *t*-Test and Wilcoxon Test Applied to Sets of Genes Classified Based on Tissue Groups and Drugs Groups

In order to determine whether the selected set of genes, *I*, are expressed distinctly between the two assigned tissue groups, *J*, {*x*_*ijkm*_|*i* ∈ *I, j* ∈ *J*}, and $\bar{J}$, $\left\{x_{ijkm}|i\in I,j\in \bar{J}\right\}$, we applied a two-way *t* test and Wilcoxon test and computed the *P*-values. Similar analyses were done with two drug groups, *K*, {*x*_*ijkm*_|*i* ∈ *I, k* ∈ *K*}, and $\bar{K}$, $\left\{x_{ijkm}|i\in I,k\in \bar{K}\right\}$.

###  Enrichment Analysis

The selected genes (gene symbols) were uploaded to Enrichr (Kuleshov et al., 2016) and Metascape (Zhou et al., 2019) in order to validate the various biological functions of the selected genes.

## Results

**Figure 2:**
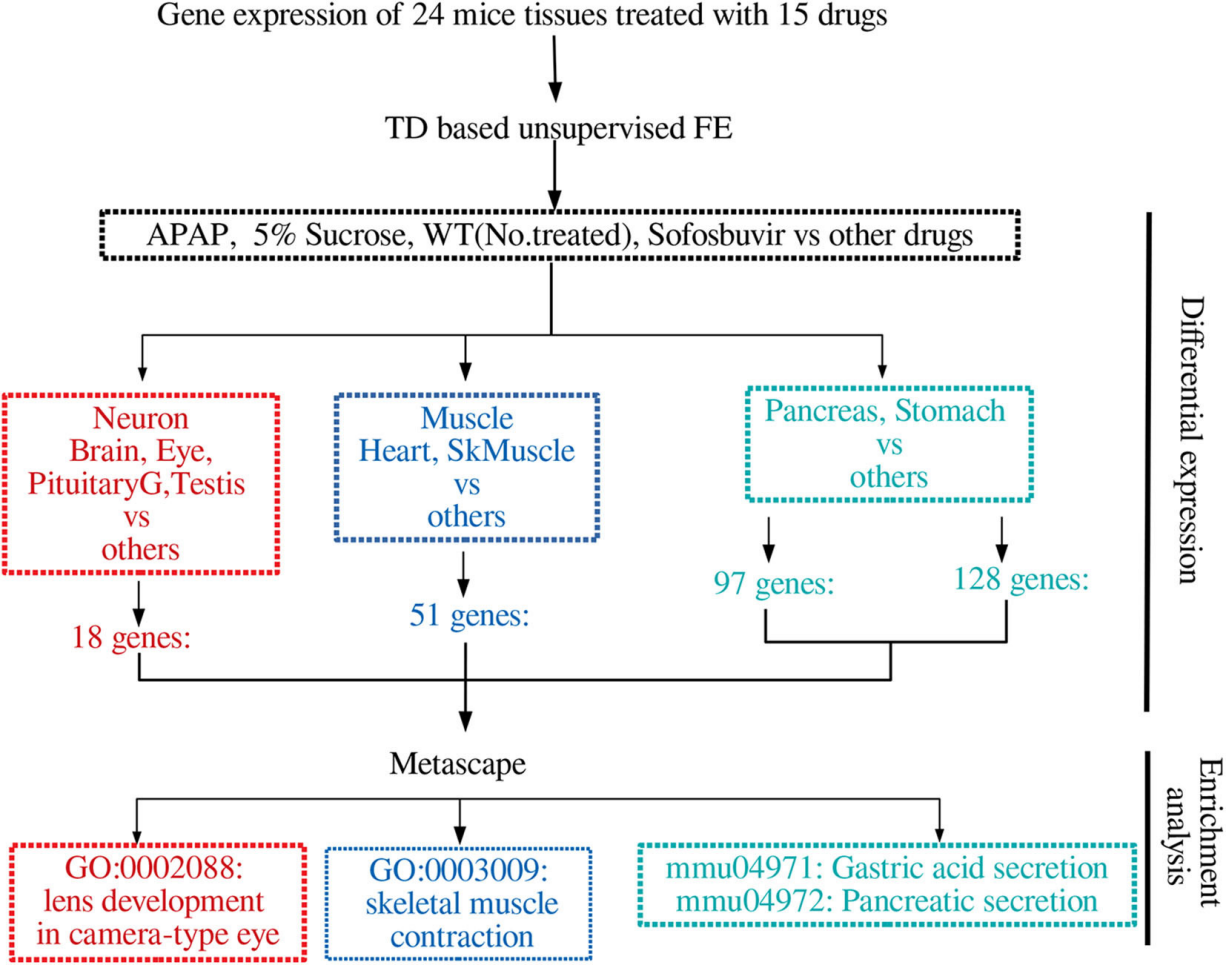
Summary of the results obtained in this study.

Figure 2 summarizes the results obtained in this study.

###  Drug Treatment Specificity

**Figure 3:**
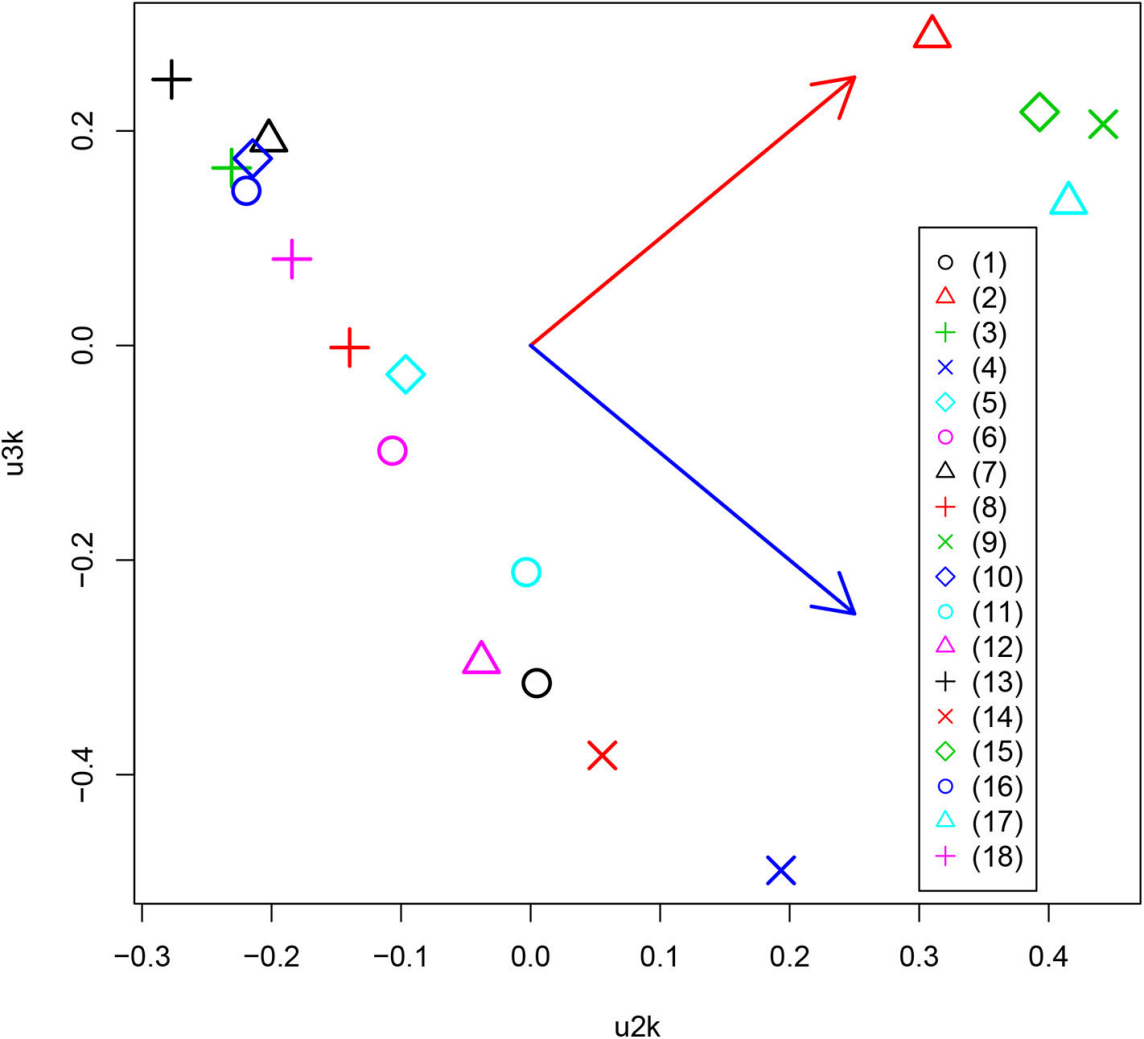
Scatter plot between *u*_2*k*_ and *u*_3*k*_ attributed to drug treatment. Red and blue arrows represent distinct controls and drug treatments, and diversity among drug treatments, respectively. (1) Alendronate, (2) APAP, (3) Aripiprazole, (4) Asenapine, (5) Cisplatin, (6) Clozapine, (7) Dox, (8) EMPA, (9) FivePercentSucrose, (10) Lenalidomide, (11) Lurasidone, (12) Olanzapine, (13) Repatha, (14) Risedronate, (15) Sofosbuvir, (16) Teriparatide, (17) WT.No.treated, (18) 5percentCMC0.25percentTween80.

After obtaining the TD, Equation (1), we first investigated *u*_ℓ_2_*k*_ attributed to the *k*th drug. Although the number of drugs tested is as many as 15, the total number of drug treatments was considered to be 18 due to the testing of three additional conditions. Usually, the first singular value vectors represent uniform values (i.e., components that are not distinct between samples) (Taguchi, 2020). In this case, *u*_1*k*_ does not represent any dependence on drug treatment. This is reasonable because the expression of most genes is unlikely to be affected by drug treatment. We thus considered the second and third singular value vectors, *u*_2*k*_ and *u*_3*k*_, attributed to drug treatments (Figure 3). In contrast to expectations, the drug treatments were quite universal. Most of the drug treatments [other than (2), (9), (15), and (17)] were separated from the control treatments [(2), (9), (15), and (17)] along one direction (red arrow) whereas the diversity among drug treatments was spread perpendicular (blue arrow) to that direction, only among drug treatments. This suggests that the gene expression profiles are altered similarly, independently of the drug treatment.

###  Tissue-Specificity

**Figure 4:**
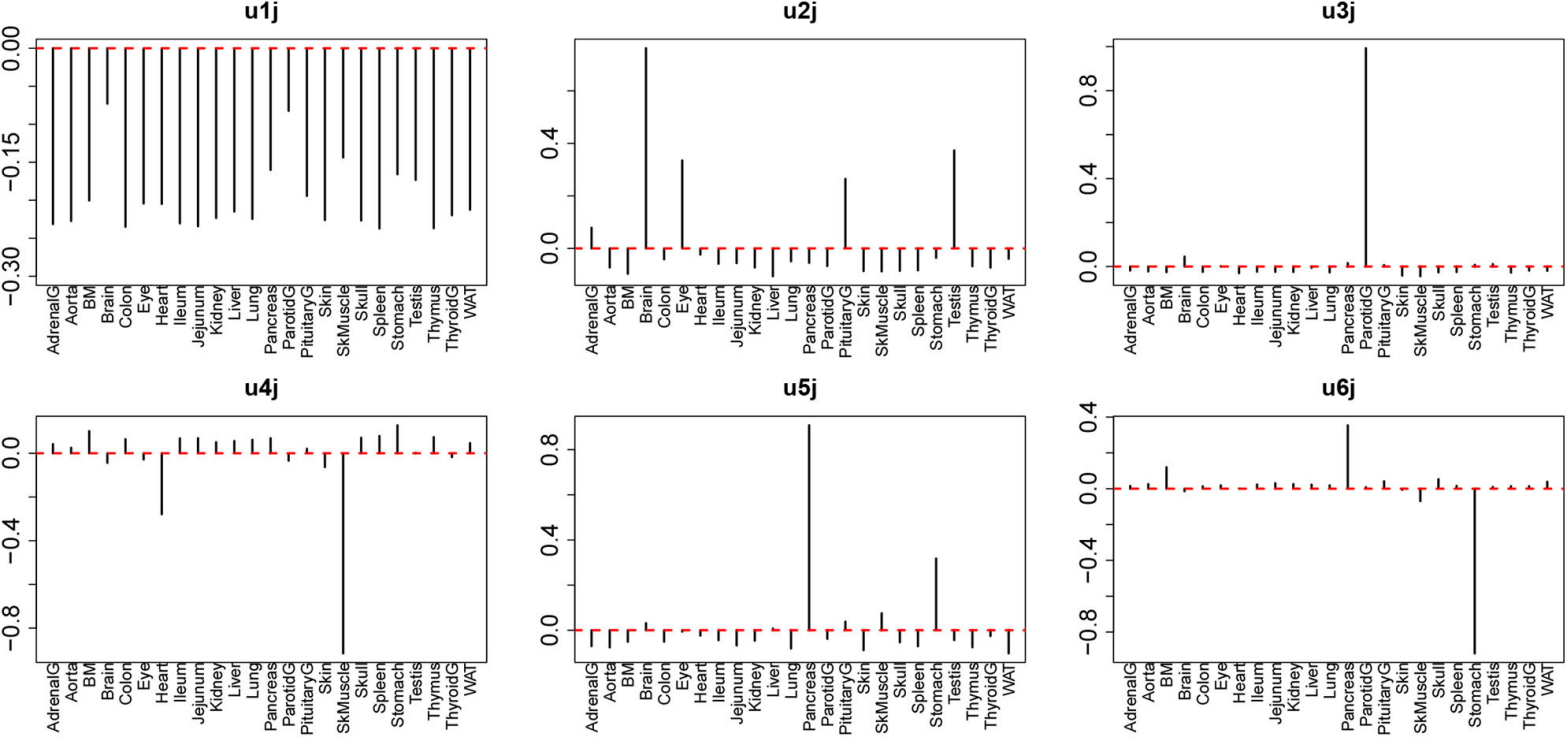
Singular value vectors, *u*_ℓ_1_*j*_, attributed to tissues. *u*_1*j*_: no tissue specificity. *u*_2*j*_: Brain Eye, Pituitary, and Testis, thus mostly neuron-specific. *u*_3*j*_: Parotid-specific, *u*_4*j*_: Heart and SkMuscle, thus muscle-specific, *u*_5*j*_ and *u*_6*j*_: stomach and pancreas, thus, gastrointestinal-specific.

We further studied the relationship of universal drug treatments with individual tissues. For this, we next investigated *u*_ℓ_1_*j*_ attributed to 24 tissues. We then found that several *u*_ℓ_1_*j*_ are expressed in a tissue-group wide manner (Figure 4). The tissue-wide expression pattern identified by singular value vectors is described as follows; As *u*_1*j*_ does not express any tissue specificities, it is unlikely to exhibit tissue specificity; as *u*_2*j*_ has larger absolute values for the brain, eye, pituitary, and testis, it is likely to represent neuronal tissue specificities; as *u*_3*j*_ has larger absolute values only for the parotid, we did not consider it further; as *u*_4*j*_ exhibits larger absolute values for the heart and SkMuscle, we considered that it exhibits muscle specificities; As *u*_5*j*_ and *u*_6*j*_ exhibit larger absolute values for the pancreas and stomach, we considered that it exhibits gastric tissue specificities. It is thus obvious that the combination of tissue specificity is quite reasonable biologically.

Aiming to specify singular value vectors attributed to genes, *u*_ℓ_4_*i*_, for gene selection, we then checked which of *G*(ℓ_1_, 2, 1, ℓ_4_) and *G*(ℓ_1_, 3, 1, ℓ_4_) have larger absolute values, as *u*_1*m*_ always exhibits the same values between two replicates (Table 1).

**Table 1:** *G*(ℓ_1_, 2, 1, ℓ_4_) and *G*(ℓ_1_, 3, 1, ℓ_4_) for ℓ_1_ = 2, 4, 5, 6.

**ℓ_1_**	**2**		**4**	
**ℓ_4_**	***G*(2, 2, 1, ℓ_4_)**	***G*(2, 3, 1, ℓ_4_)**	***G*(4, 2, 1, ℓ_4_)**	***G*(4, 3, 1, ℓ_4_)**
1	131.248442	19.7819438	−98.4349019	−13.498228
2	**−173.243689**	**−23.9915660**	−4.8528076	1.113899
3	−11.859736	−3.2551088	−0.1595594	−1.116396
4	13.669561	2.4373120	**−81.3734282**	**36.838277**
5	26.610843	−0.3136913	−22.2440356	9.820737
6	−1.275395	4.5339065	−1.3753621	−5.318282
7	−18.306263	15.9791077	21.3673134	−11.230437
8	20.891762	26.5918473	3.9733331	−7.152480
9	21.836494	16.1461476	9.2972447	2.232529
10	11.717415	−12.8960548	1.4137802	−7.748038
**ℓ_1_**	**5**		**6**	
**ℓ_4_**	***G*(5, 2, 1, **ℓ**_4_)**	***G*(5, 3, 1, **ℓ**_4_)**	***G*(6, 2, 1, **ℓ**_4_)**	***G*(6, 3, 1, ℓ_4_)**
1	97.897860	−42.9481806	72.181307	27.3218396
2	9.267391	4.5503920	3.780984	6.3881436
3	−3.744432	0.2003586	2.340165	−0.2130656
4	1.648558	3.4031386	−9.812308	2.8751439
5	**93.027741**	**−56.9322793**	6.435061	8.5776220
6	−57.463765	23.2247109	**−19.332916**	**34.1868710**
7	28.276681	−26.9479131	**30.604535**	**−18.8319412**
8	12.884351	−13.8270607	1.798188	−10.6484624
9	−5.865058	1.0216563	9.581512	0.3507831
10	15.683762	3.7893181	−14.429706	−4.7985105

*Values in bold correspond to those of ℓ_4_s used for gene selection with u_ℓ_4_i_*.

For ℓ_1_ = 2, which is supposed to be attributed to neuron-specific tissues (*u*_2*j*_), *G*s with ℓ_4_ = 2 have larger absolute values. Thus, *u*_2*i*_ was employed for neuron-specific gene selection. For ℓ_1_ = 4, which is supposed to be attributed to muscle-specific tissues (*u*_4*j*_), *G*s with ℓ_4_ = 4 have larger absolute values. Thus, *u*_4*i*_ was employed for muscle-specific gene selection. For ℓ_1_ = 5, which is supposed to be attributed to gastrointestinal-specific tissues (*u*_5*j*_), *G*s with ℓ_4_ = 5 have larger absolute values. Thus, *u*_5*i*_ was employed for muscle-specific gene selection. For ℓ_1_ = 6, which is also supposed to be attributed to gastrointestinal-specific tissues (*u*_6*j*_), *G*s with ℓ_4_ = 6, 7 have larger absolute values. Then, *u*_6*i*_ and *u*_7*i*_ were employed for muscle-specific gene selection.

**Figure 5:**
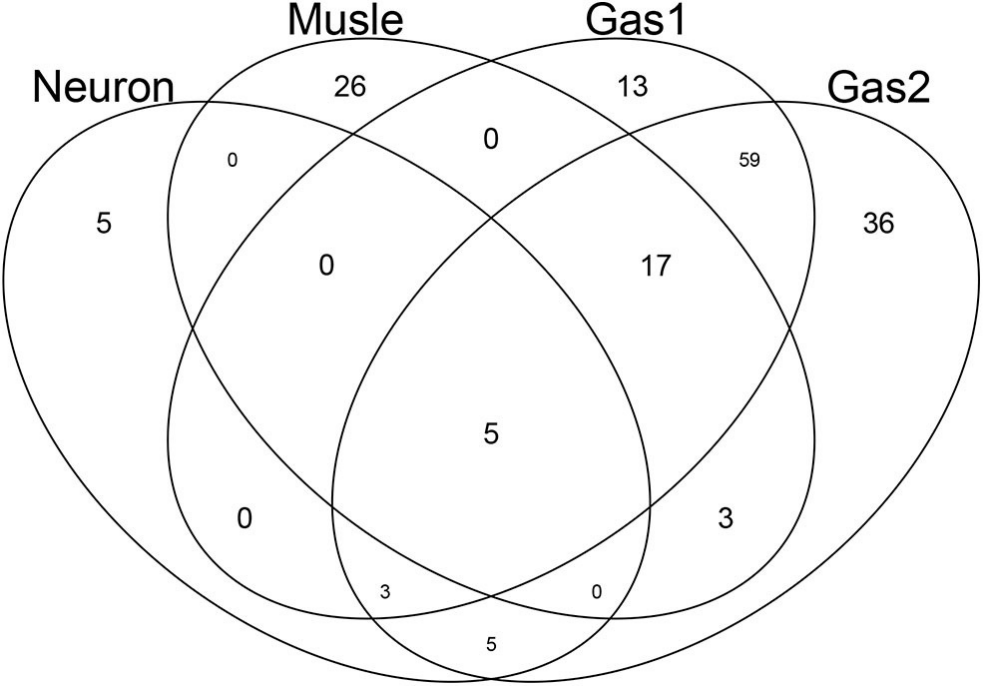
Venn diagram of genes selected by TD-based unsupervised FE. Neuron: genes associated with *u*_2*j*_, which is supposed to be neuron-specific. Muscle: genes associated with *u*_4*j*_, which is supposed to be muscle-specific. Gas1 and Gas2: genes associated with *u*_5*j*_ and *u*_6*j*_, respectively, which are supposed to be gastrointestinal-specific.

After computing the adjusted *P*-values, *P*_*i*_, attributed to the genes (see methods), the genes associated with adjusted *P*_*i*_ < 0.01 were selected (Table 2). The lists of selected genes can be found in supporting information (Additional File 1). Figure 5 shows a Venn diagram of the selected genes. As expected, two sets of genes, Gas1 and Gas2, which are supposed to be gastrointestinal-specific, are quite common. Other than these, the selected genes are quite distinct from one another. Thus, TD-based unsupervised FE successfully identified the genes whose expression was affected by the drugs in a tissue group-specific manner.

**Table 2:** Statistical tests for distinct expression between the specified tissues and other tissues, and between drug treatments and controls.

				****P****-**values by statistical tests**		
				**Tissues**	**Drug treatment**	
**ℓ_1_**	**Tissue specificity**	**# of Genes**	**Specified tissues**	****t**-test**	**Wilcoxon test**	***t*-test**
2	Neuron	18	Brain, Eye, Pituitary, Testis	2.14 × 10^−24^	9.65 × 10^−49^	0.22
4	Muscle	51	Heart, SkMuscle	1.99 × 10^−55^	2.67 × 10^−77^	0.04
5	Gastrointestinal	97	Pancreas, Stomach	8.48 × 10^−11^	2.73 × 10^−40^	8.13 × 10^−22^
6		128		6.67 × 10^−8^	8.69 × 10^−90^	8.69 × 10^−90^

###  Confirmation of Differential Expression

In order to check whether the selected genes are expressed distinctly between the specified tissues and other tissues, as well as between drug treatments and controls, we first applied statistical tests to the selected genes (Table 2). The data clearly showed that for all cases, gene expression was distinct between the specified tissues and other tissues as well as between drug treatments and controls. Thus, TD-based unsupervised FE allowed us to select the genes whose expression is coincident with *u*_ℓ_2_*k*_s in Figure 3 and *u*_ℓ_1_*j*_s in Figure 4.

###  Biological Evaluation

**Figure 6:**
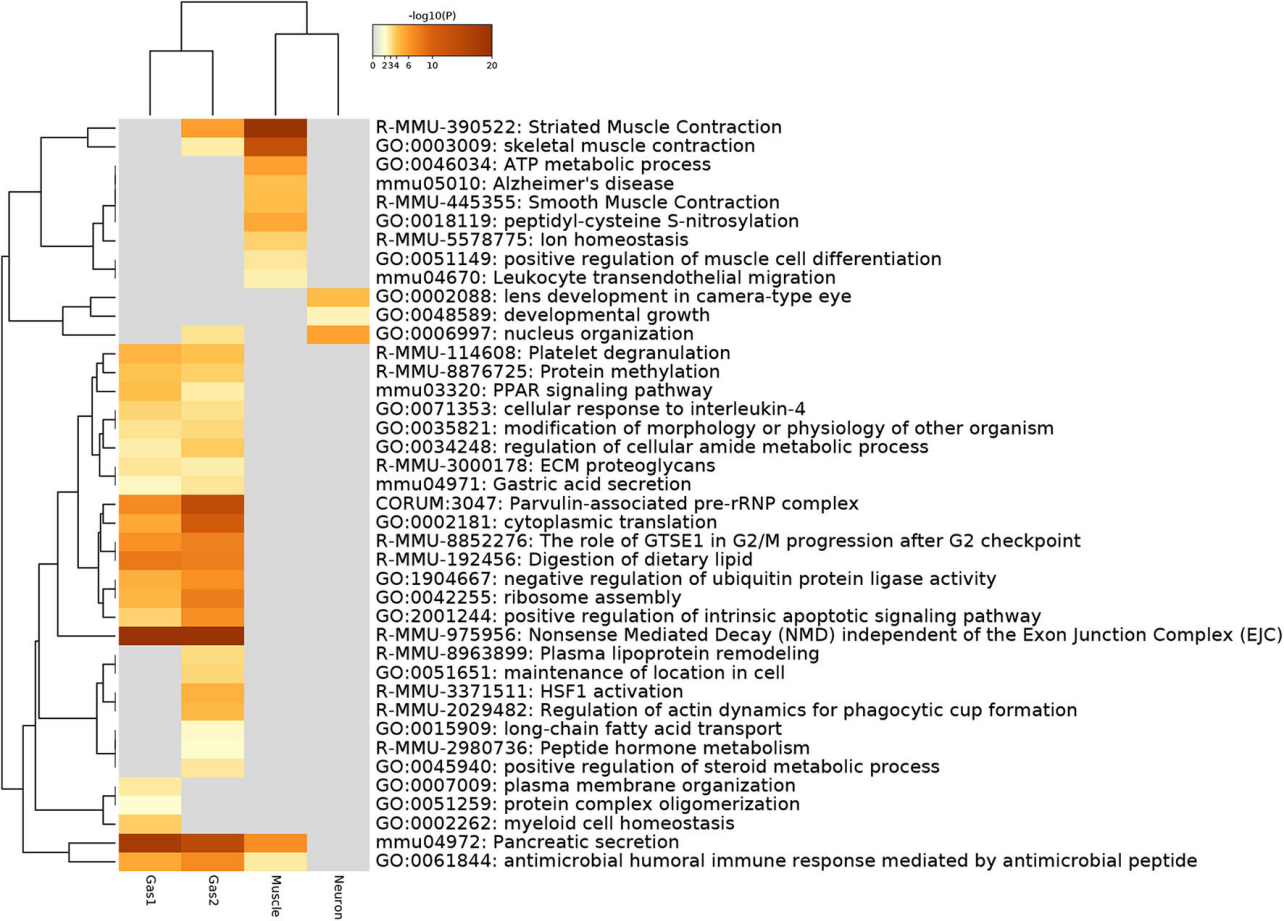
Heatmap of enrichment analysis provided by Metascape.

Next, we evaluated the selected genes biologically. For this purpose, we first uploaded the genes to Metascape (Figure 6). Initially, we noticed that Gas1 and Gas2 largely shared the enriched terms as expected, even though these two gene sets were selected using distinct singular values (*u*_5*i*_ and *u*_6*i*_, *u*_7*i*_, respectively). In particular, it is important to note that two KEGG terms, “mmu04971: Gastric acid secretion” and “mmu04972: Pancreatic secretion” are shared by Gas1 and Gas2, which are supposed to be Pancreas- and Stomach-specific. In contrast, various muscle-related terms are enriched in the Muscle gene set as expected, whereas “GO:0002088: lens development in camera-type eye” is enriched in the neuronal gene set. All of these results suggest that TD-based unsupervised FE selected the biologically reasonable genes.

**Figure 7:**
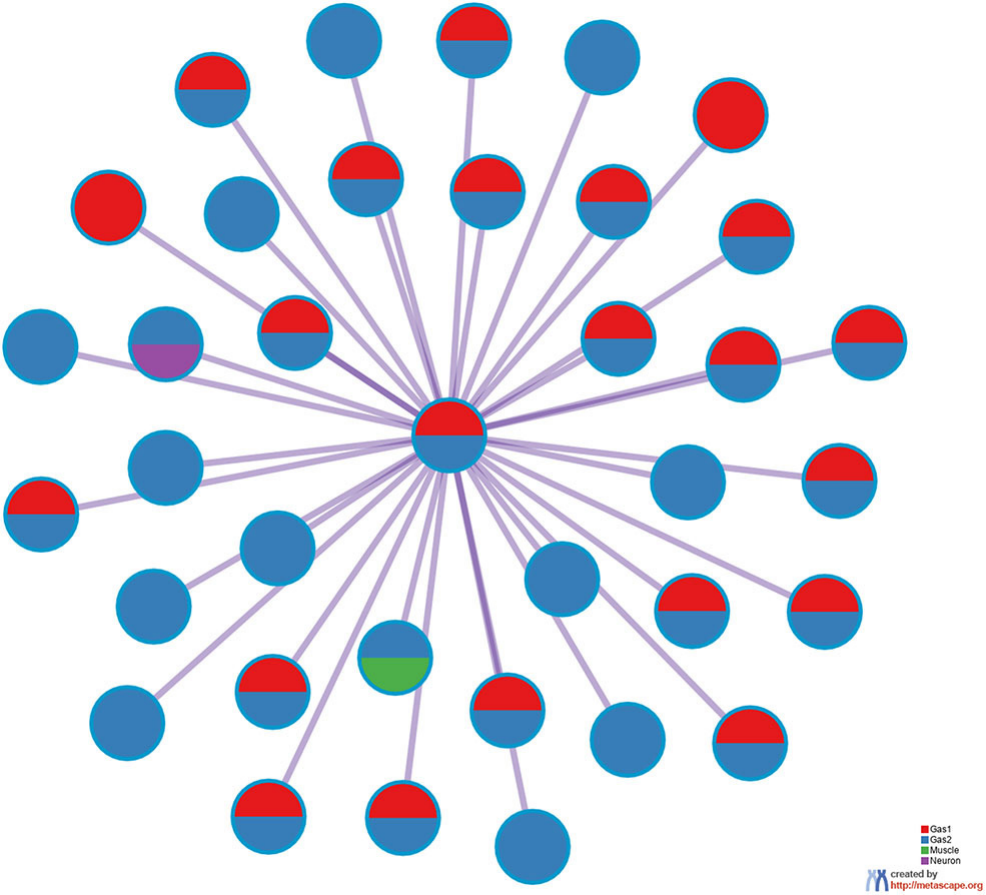
PPI network provided by Metascape. Red: Gas1, Blue: Gas2, Green: Muscle, Purple: Neuron.

Figure 7 shows the protein-protein interaction (PPI) network provided by Metascape. A high degree of connectivity was obvious. Thus, TD-based unsupervised FE identified the sets of genes among which PPI is enriched. Moreover, Gas1 and Gas2 largely share the PPI network, whereas the neuronal and muscular gene sets form their own PPI network within which PPI is enriched. Thus, PPI analysis also indicated that TD-based unsupervised FE identified biologically reasonable genes.

To eliminate the possibility that our results were specific to the Metascape data set, we uploaded the genes selected by TD-based unsupervised FE to Enrichr (Table 3). With this data set, we observed clear detection of at least one tissue-related disease for four sets of tissue-specific genes, validating the Metascape-based results.

**Table 3:** Enrichment analysis for “Disease Perturbations from GEO up” and “Disease Perturbations from GEO down” by Enrichr.

**Disease perturbations from GEO up**			
**Term**	**Overlap**	***P*-value**	**Adjusted *P*-value**
**Neuron-specific genes**			
**Amyotrophic lateral sclerosis** DOID-332 mouse GSE3343 sample 685	5/138	1.16 × 10^−7^	9.72 × 10^−5^
**Retinitis Pigmentosa** C0035334 mouse GSE128 sample 33	5/338	9.57 × 10^−6^	4.01 × 10^−2^
**Muscle specific**			
Polycystic Ovary Syndrome C0032460 human GSE6798 sample 292	21/306	2.87 × 10^−25^	2.41 × 10^−22^
Polycystic ovary syndrome DOID-11612 human GSE8157 sample 880	21/325	1.03 × 10^−24^	4.32 × 10^−22^
Insulin Resistance DOID-9352 human GSE36297 sample 581	20/290	4.52 × 10^−24^	1.26 × 10^−21^
**Neurogenic Muscular Atrophy** C0270948 rat GSE2566 sample 396	18/208	1.98 × 10^−23^	4.15 × 10^−21^
**Nemaline myopathy** DOID-3191 mouse GSE3384 sample 976	15/150	1.65 × 10^−20^	2.77 × 10^−18^
Psoriasis DOID-8893 mouse GSE27628 sample 822	18/346	2.06 × 10^−19^	2.88 × 10^−17^
**Nemaline Myopathy** C0206157 mouse GSE3384 sample 160	16/276	5.20 × 10^−18^	6.24 × 10^−16^
**Muscular Dystrophy** C0026850 mouse GSE2507 sample 405	16/278	5.84 × 10^−18^	6.13 × 10^−16^
Cystic fibrosis DOID-1485 mouse GSE3100 sample 1057	17/344	5.91 × 10^−18^	5.51 × 10^−16^
COPD - Chronic obstructive pulmonary disease C0024117 human GSE475 sample 343	16/289	1.09 × 10^−17^	9.11 × 10^−16^
**Disease perturbations from GEO down**			
**Term**	**Overlap**	***P*****-value**	**Adjusted** ***P*****-value**
**Gas1 genes**			
**Pancreatitis** DOID-4989 mouse GSE3644 sample 513	36/238	9.21 × 10^−45^	7.73 × 10^−42^
Skin squamous cell carcinoma DOID-3151 human GSE2503 sample 627	37/373	5.24 × 10^−39^	2.20 × 10^−36^
**Pancreatic ductal adenocarcinoma** DOID-3498 mouse GSE53659 sample 699	26/101	1.24 × 10^−38^	3.48 × 10^−36^
**Pancreatic invasive intraductal papillary-mucinous carcinoma** DOID-8150 human GSE19650 sample 610	31/248	1.21 × 10^−35^	2.54 × 10^−33^
Cystic fibrosis DOID-1485 mouse GSE769 sample 1058	32/288	3.92 × 10^−35^	6.58 × 10^−33^
**Acute pancreatitis** C0001339 mouse GSE3644 sample 376	28/188	2.33 × 10^−34^	3.26 × 10^−32^
Cystic Fibrosis C0010674 mouse GSE769 sample 428	31/275	3.34 × 10^−34^	4.00 × 10^−32^
Chronic phase chronic myelogenous leukemia DOID-8552 human GSE5550 sample 456	30/270	7.05 × 10^−33^	7.40 × 10^−31^
Invasive ductal carcinoma DOID-3008 human GSE21422 sample 606	31/304	8.09 × 10^−33^	7.54 × 10^−31^
Eczema C0013595 human GSE6012 sample 268	26/163	1.06 × 10^−32^	8.87 × 10^−31^
**Gas2 genes**			
Skin squamous cell carcinoma DOID-3151 human GSE2503 sample 627	51/373	9.37 × 10^−55^	7.86 × 10^−52^
**Pancreatitis** DOID-4989 mouse GSE3644 sample 513	45/238	1.14 × 10^−54^	4.77 × 10^−52^
Systemic lupus erythematosus DOID-9074 human GSE10325 sample 691	43/210	9.36 × 10^−54^	2.62 × 10^−51^
Systemic lupus erythematosus (SLE) DOID-9074 human GSE36700 sample 512	47/294	1.50 × 10^−53^	3.15 × 10^−51^
Invasive ductal carcinoma DOID-3008 human GSE21422 sample 606	44/304	5.90 × 10^−48^	9.90 × 10^−46^
Eczema C0013595 human GSE6012 sample 268	37/163	7.47 × 10^−48^	1.04 × 10^−45^
Malignant Melanoma C0025202 human GSE3189 sample 117	41/250	6.75 × 10^−47^	8.09 × 10^−45^
Chronic phase chronic myelogenous leukemia DOID-8552 human GSE5550 sample 456	41/270	1.91 × 10^−45^	2.00 × 10^−43^
Sickle Cell Anemia C0002895 human GSE9877 sample 109	37/197	1.61 × 10^−44^	1.50 × 10^−42^
Actinic keratosis C0022602 human GSE2503 sample 350	46/429	4.69 × 10^−44^	3.94 × 10^−42^

*Diseases in bold correspond to those related to specific tissues. Up to the top 10 ranked terms are shown*.

## Discussion

Although it is unclear why the 15 drugs affected the expression of many common genes, a detailed investigation can allow further interpretation. Table 4 shows the drugs' effects on neuronal, muscular, and pancreatic tissues. These data suggest that most drugs simultaneously affect these three groups of tissues.

**Table 4:** Previously reported drug effects on neuron (brain and eye), muscle and pancreas tissues.

	**Tissue types**		
**Drugs**	**Neuron**	**Muscle**	**Pancreas or stomach**
Alendronate	Brain calcification (Oliveira and Oliveira, 2016)	Muscle mass (Harada et al., 2015)	Pancreatitis (Hung, 2014)
Acetaminophen (APAP)	Brain (Ghanem et al., 2016)	Skeletal muscle (Trappe et al., 2011)	Pancreatitis (Chen et al., 2015)
Aripiprazole	Brain activation (Myrick et al., 2010)	Muscle spasms (^*^)	Pancreatitis (Kiraly and Gunning, 2008)
Asenapine	Cognitive and monoamine dysfunction Elsworth et al. (2012)	Muscle rigidity(^*^)	—
Cisplatin	Prefrontal cortex (Huo et al., 2018)	Muscle atrophy (Sakai et al., 2014)	Pancreas (Yadav, 2019)
Clozapine	Brain (Li et al., 2014)	Myotoxicity (Reznik et al., 2000)	Pancreatitis (Bergemann et al., 1999)
Doxycycline	Brain (Lucchetti et al., 2018)	Smooth Muscle (Bendeck et al., 2002)	Acute pancreatitis (Rawla and Raj, 2017)
Empagliflozin	Neurovascular unit and neuroglia (Hayden et al., 2019)	Muscle sympathetic nerve activity (Jordan et al., 2017)	Pancreatitis (Kishimoto et al., 2019)
Lenalidomide	Memory loss (Rollin-Sillaire et al., 2013)	Muscle cramp (Reece et al., 2012)	Pancreatic cancer (Ullenhag et al., 2017)
Lurasidone	Acute schizophrenia (Yasui-Furukori, 2012)	Muscle (^*^)	—
Olanzapine	Brain stem (Anwar et al., 2016)	Acute muscle toxicity (Keyal et al., 2017)	Pancreatitis (Kerr et al., 2007)
Repatha (Evolocumab)	—	Muscle-related statin Intolerance (Nissen et al., 2016)	—
Risedronate (actonel)	Ocular myasthenia (Raja et al., 2007)	Muscle weakness (Badayan and Cudkowicz, 2009)	Gastrointestinal cancer (Vinogradova et al., 2013)
Sofosbuvir	Ocular surface (Salman, 2016)	Myositis (Patel et al., 2015)	Pancreatitis (Margapuri and Jubbal, 2019)
Teriparatide	—	Muscle cramp (Kakaria et al., 2005)	Pancreatitis (^*^)

* *Reported side effects*.

Our results are in contrast to the study that inspired our work (Kozawa et al., 2020), in which the authors employed a fully supervised approach requiring previous knowledge. Although Kozawa et al. (2020) also aimed to infer the therapeutic and side effects of drug treatments in humans based on gene expression in drug-treated tissues, their analysis required previous knowledge that is not needed for TD-based unsupervised FE. In this sense, our approach has distinct potential that the original study could not achieve.

In addition to the above-mentioned biological superiority of TD-based unsupervised FE, this approach also has some methodological advantages as follows. First, although we classified 24 tissues into two groups based on the observation of singular value vectors attributed to tissues, *u*_ℓ_1_*j*_ (Figure 4) prior to the identification of differentially expressed genes, it is computationally infeasible for other methods to classify 24 tissues into two groups before starting to seek differentially expressed genes, as there are no criteria on how to divide 24 tissues into two groups. It is thus practically impossible to analyze all possible divisions, as they number in the millions. The same advantage is observed when grouping 18 drug treatments into two. This may be much easier than classifying tissues, because some of the drug treatments are obviously controls. Nevertheless, based upon the second and third singular value vectors attributed to drug treatments, *u*_2*k*_ and *u*_3*k*_ (Figure 3), acetaminophen (APAP) and sofosbuvir are grouped together with two control treatments. Such a classification can never be proposed without TD. In this sense, there is no computationally feasible method that can compete with our method.

The biological basis for the two groups of drugs seen in Figure 3 may be questioned. To clarify this point, we uploaded two groups of drugs to DrugEnrichr (Kuleshov et al., 2020), which evaluates the coincidence of genes targeted by the uploaded drugs (Additional File 2). Based on the “Geneshot Predicted from Co-expression” category in DrugEnrichr, we found that there are at least as many as 164 genes targeted by two drugs (APAP and Sofosbuvir) in group1 whereas 213 genes are targeted by at least two drugs among as many as 13 drugs included in group2 (Alendronate, Aripiprazole, Asenapine, Cisplatin, Clozapine, Dox, EMPA, Lenalidomide, Lurasidone, Olanzapine, Repatha, Risedronate, Teriparatide). This suggests that these two groups of drugs are quite distinct because there are no common targeted genes between these 164 and 213 genes. Thus, the groups of drugs identified by TD based unsupervised FE are likely based on the genes that the drugs target.

In view of the two above-mentioned advantages, TD-based unsupervised FE might yield completely distinct outcomes that other supervised methods cannot, and it therefore represents a worthwhile primary or supplementary approach to gene-expression-based investigation of drug effects.

One might wonder if the results were confirmed only by single experiments. As the results shown in Table 3 indicate coincidence between the present result and other studies, the results derived in this study are not dependent on a single study, but are coincident with numerous studies in the public domain database.

Moreover, TD-based unsupervised FE is a very useful strategy for repositioning known drugs. As shown in Figure 3, TD-based unsupervised FE can determine the effective tissue. Furthermore, as indicated in Table 3, the genes selected by TD-based unsupervised FE can indicate the diseases for which the drugs have potential effectiveness. Therefore, applying TD-based unsupervised FE to gene expression profiles altered by drug treatments can be a promising strategy to repurpose known drugs for new diseases.

## Conclusions

In this paper, we applied TD-based unsupervised FE (Taguchi, 2020) to the gene expression profiles of 24 mouse tissues treated with 15 drugs. Integrated analysis allowed us to identify the universal nature of drug treatments in a tissue-group-wide manner, which is generally impossible to identify using any other supervised strategy that requires prior information.

## Data Availability Statement

All datasets analyzed in this study were obtained from GEO: GSE142068.

## Author Contributions

Y-hT planned and performed the study. Y-hT and TT discussed the results and wrote the paper. All authors contributed to the article and approved the submitted version.

## Conflict of Interest

The authors declare that the research was conducted in the absence of any commercial or financial relationships that could be construed as a potential conflict of interest.

## Supplementary Material

Additional File 1 List of genes selected by TD-based unsupervised FE. List of genes shown in Table 2 (xlsx).

Additional File 2 List of genes predicted by DrugEnrichr, whose expression is likely affected by the drugs investigated in this study. List of genes (xlsx).
